# Evaluation of Masticatory Forces in Patients Treated for Mandibular Fractures: A Case-Control Study

**DOI:** 10.7759/cureus.29295

**Published:** 2022-09-18

**Authors:** Shrutika M Salunkhe, Harshawardhan Kadam, Manisha Nakhate, Effie Edsor, Ranjit Kamble, Ashvini Kishor Vadane

**Affiliations:** 1 Department of Oral and Maxillofacial Surgery, Dr. D. Y. Patil Dental College and Hospital, Dr D. Y. Patil Vidyapeeth, Pune, IND; 2 Department of Oral and Maxillofacial Surgery, Bharatividyapeeth Dental College and Hospital, Sangli, IND; 3 Department of Anatomy, Dr. DY Patil Medical College, Nerul, IND; 4 Department of Oral and Maxillofacial Surgery, Sree Mookambika Institute of Dental Sciences, Kulasekaram, IND; 5 Department of Orthodontics, Sharad Pawar Dental College & Hospital, Datta Meghe Institute of Medical Sciences, Wardha, IND; 6 Department of Oral and Maxillofacial Surgery, M.A. Rangoonwala College of Dental Sciences and Research Centre, Pune, IND

**Keywords:** bilateral fracture, unilateral fracture, functional masticatory force, mandibular fractures, bite force measuring device

## Abstract

*Purpose:*​​​​​ This study aimed to evaluate the masticatory forces in patients treated for mandibular fractures. To assess the magnitude of damage to the masticatory system caused by the various mandibular fractures and the period required for their normalization.

*Materials and Methods:* Data were recorded from the authentic and original bite force measurement device from 2015 to 2017. The sample was composed of 30 isolated mandible fractures patients, Group 1 consisting 15 patients with unilateral mandible fractures, Group 2 consisting 15 patients with bilateral mandible fractures, treated with ORIF (open reduction immobilization fixation) under general anesthesia, and Group 3 was a control group. Predictor variables were drawn from predefined intervals for three months (ninth POW) postoperative week.

The condition of wound healing was checked, and masticatory forces are measured at the first, fourth, sixth, and ninth postoperative weeks and compared with a control group of the same age and gender. The outcome variables were the success rate and associated complications.

*Results:* For the study, a total of 30 patients with 15 bilateral and 15 unilateral isolated mandibular fractures were included. The study result suggests that the patient had lower bite forces relative to the control group at all intervals. All patients showed a significant increase in the bite force values from the first to the fourth postoperative weeks (p = ‹0.001), which also increased significantly from the fourth to sixth postoperative weeks (p = ‹0.001), and from the sixth to the ninth week.

*Conclusion:* ​​​​ Based on the results obtained from our study, we conclude that there is a temporary adverse effect on masticatory forces. Fracture of the bilateral mandible has a stronger influence on bite force than unilateral mandible fracture. These fractures also take a longer time to normalize.

## Introduction

The maxillofacial zone is a common site for traumatic injuries, which has a direct impact on facial aesthetics and function in patients. The mandible is the main structural skeletal bone related to the face, and the maxillofacial region is a common site for these types of injuries. [1]. So, understanding the mandible's bio-mechanics is important as it involves many functions. The prognosis of fracture treatment is to rectify the endurance of the fracture site to normal and attain regular masticatory function [2]. Chewing work defines the capacity of the suspect to bite painlessly. The main factors of chewing functions were the frequency of functional movement of the mandible, bite, increased bite force, and actions of chewing muscles [3].

Champy technique is a well-accepted technique as it involves the placement of monocortical plates along the ideal line of osteosynthesis and which allows an early return of function and minimal invasiveness [4]. The treatment, which consists of surgery along the fracture in the mandible, focuses on the restoration of the structural shape of the mandible, with the expectation that both normal forms and functions would be restored [5]. In the process of any such surgical treatment, injury in the form of slicing of chewing muscles, the resultant soft tissue, and unknown cause nerve injury can further affect the chewing system [6].

Though current CT (Computed Tomography) imaging modalities like 2D/3D views are capable of providing detailed information on internal and external anatomic body features. But we cannot assume the impact of mandible fracture on masticatory force with the help of this technique [7].

An increase in bite forces is the main parameter of chewing function and also are very delicate to estimate and interpret [8]. The masticatory force measurement is the key factor to measure the time of the establishment of function. The study was based on the time required for the establishment of maximum masticatory force. This method was used to learn about patients with maxillofacial defects during preoperative and postoperative surgical correction [9]. Since an increased bite force is minimized with fractures within the chewing system, bite direction must be regained after surgery. There was an iatrogenic final difference in the alteration of tissue structures of the chewing apparatus as the person effected might be able to show bite loads [10].

In our study, we are recording maximum masticatory force at an interval of the first, fourth, sixth, and ninth postoperative week. It is compared with a voluntary control group of the same age and gender in the Indian population.

## Materials and methods

The Institutional Ethical Committee of Bharati Vidyapeeth Deemed University Medical College & Hospital, Sangli, approved this study as well as the participants' signed consent agreement with ethical number BVDUMC&H/ Sangli/ IEC/ Dissertation 2015-16/151. The study includes patients treated for mandibular fractures in college from October 2015 to September 2016.

**Figure 1:**
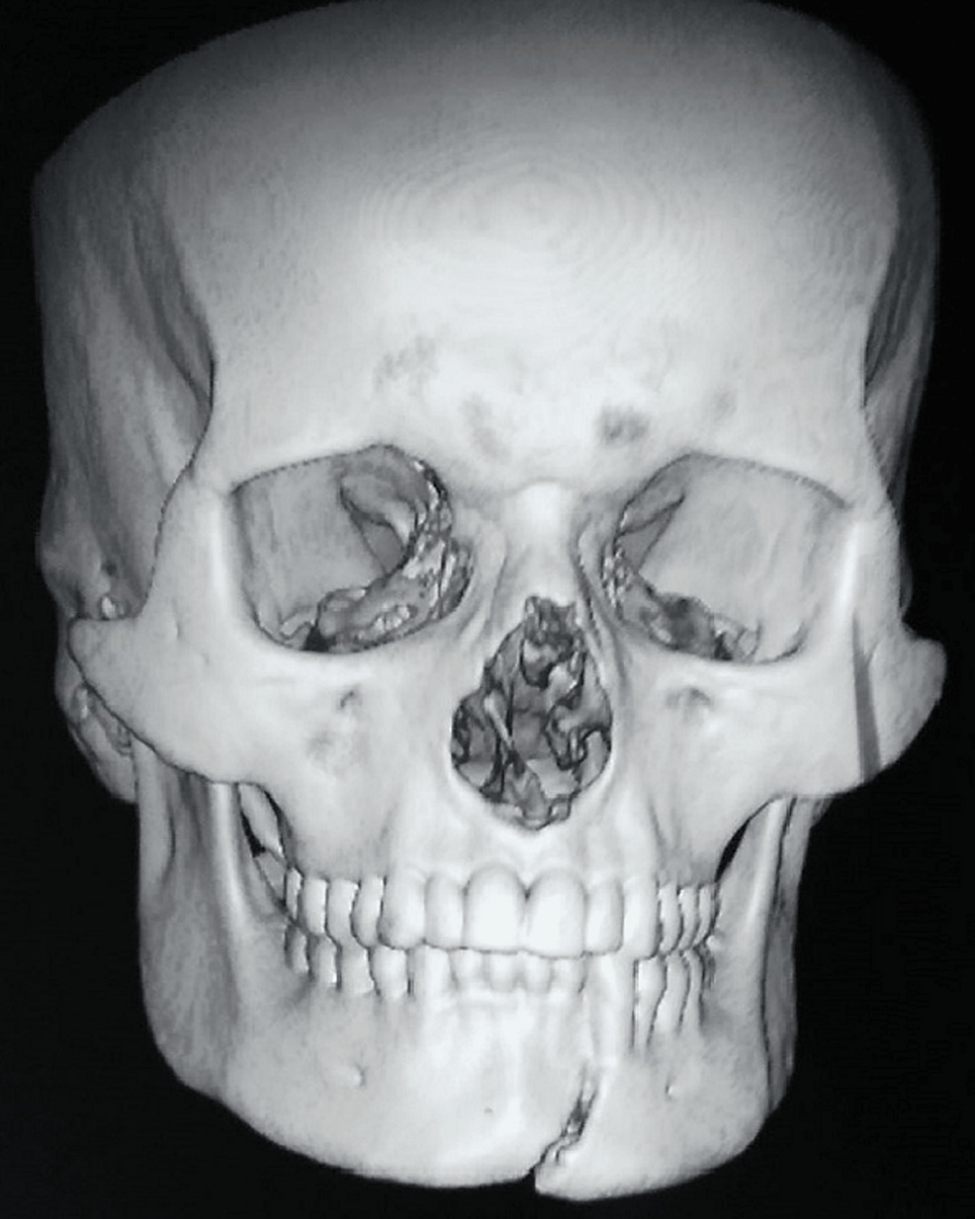
The fracture lines studied

Thirty patients were equally divided according to their fracture pattern into unilateral fracture and bilateral fracture of the mandible. According to Dingman and Natvig’s classification of mandible fractures by anatomic region in the unilateral mandible fracture, we included symphysis and parasymphysis, and angle fracture cases. In bilateral mandible fracture, we included ipsilateral parasymphysis and contra lateral Subcondyle or angle or bilateral parasymphysis fracture (Figure 1).

The exclusion criteria were: (1) patients with associated mid-face fractures, (2) edentulous patients, (3) patients below 14 years of age, and (4) medically compromised patients.

Case selection inclusion criteria include patients between the ages of 17 and 50, patients undergoing displacement and reduction, medically fit patients who are operable, dentulous and adult patients, patients associated with isolated mandible fractures, and patients who are ready for follow-up. Voluntary group inclusion criteria include same age and gender. The volunteers were also medically fit with bilaterally intact first molars.

All preoperative detailed medical examinations were done. The site of the incision was infiltrated with adrenaline. In mandibular fractures, all cases with lower vestibular incision and condyle fractures with an extraoral approach were given. Open reduction to achieve anatomic reconstruction and immobilization. Fixation was done with a 2 mm miniplate stainless steel plate, a four-hole gap, and two 8 mm stainless steel screws. The closure is achieved with 3-0 vicryl. Follow-up was done at predefined intervals for three months. The condition of wound healing was checked, and masticatory forces were measured on the first, fourth, sixth, and ninth post-surgery days. The standardized occlusal load measurement device Dentoforce 2 (ITL AB, Sollentuna, Sweden) was used.

All measurements were recorded on the authentic and original device. Statistical analysis was done by using SPSS Inc. Released 2007. SPSS for Windows, Version 16.0. Chicago, SPSS Inc. The data were analyzed, and results were produced using mean, standard deviation, and student's "t" tests (unpaired test and paired test).

## Results

Mandibular fractures have a significant impact on masticatory function. A major cause can also be the affected patient’s ability to chew forcefully in obtaining subnormal forces. This is related to both the comfort of the dentition and mental attitude. Injury to soft components and maxillofacial structures in the face region was assessed. Some patients were scared to move their jaw generously, especially in the first two to three weeks [5]. Table 1 shows a comparison of control and ninth postoperative week groups with Bilateral Mandibular Fractures by unpaired t-test.

**Table 1:** Comparison of control and ninth postoperative week groups with Bilateral Mandibular Fractures by unpaired t-test POW: Postoperative week, SD: Standard deviation, SE: Standard error

^Groups ^	^Mean^	^SD^	^SE^	^t-value^	^P-value^
^Ninth POW^	^251.27^	^22.96^	^5.93^		
^Control group^	^262.40^	^25.24^	^6.52^	^-1.2637^	^0.2168^

The study results suggest that patients had lower bite forces relative to the control group at all intervals. But patients' bite force values from six weeks gradually recovered. The results obtained in our study showed a difference in the restoration of maximum bite force with respect to the location of fractures in the mandible. Based on this difference, we would like to discuss the result of our study by dividing our patients into two groups. Group 1 consists of unilateral mandibular fractures. Patients in Group 2 have bilateral mandible fractures. In Group 1, we found that chew force filling was normal by the ninth postoperative week (Table 2).

**Table 2:** Comparison of bilateral mandibular fractures on various postoperative weeks and control by student's paired t-test POW: postoperative week

POW	Mean	Standard deviation	Mean Difference	Standard Difference	Percentage of change	Paired t	P-value
First POW	132.73	16.58					
Fourth POW	164.33	16.05	-31.60	23.85	-23.81	-5.1315	<0.001
First POW	132.73	16.58					
Sixth POW	187.60	20.98	-54.87	25.51	-41.34	-8.3304	<0.001
First POW	132.73	16.58					
Ninth POW	251.27	22.96	-118.53	28.75	-89.30	-15.9653	<0.001
Fourth POW	164.33	16.05					
Sixth POW	187.60	20.98	-23.27	15.08	-14.16	-5.9763	<0.001
Fourth POW	164.33	16.05					
Ninth POW	251.27	22.96	-86.93	21.25	-52.90	-15.8430	<0.001
Sixth POW	187.60	20.98					
Ninth POW	251.27	22.96	-63.67	29.09	-33.94	-8.4778	<0.001

The reason for the restoration of bite force is linked to the very small contents of the chewing apparatus. Table 3 shows a comparison of control and ninth postoperative week groups with unilateral mandibular fractures by unpaired t-test.

**Table 3:** Comparison of control and ninth postoperative week groups with unilateral mandibular fractures by unpaired t-test POW: Postoperative week, SD: Standard deviation, SE: Standard error

Groups	Mean	SD	SE	t-value	P-value
Ninth POW	230.00	18.78	4.85		
Control group	241.07	20.49	5.29	-1.5418	0.1343

In Group 2, bilateral fractures of the mandible were restored; bite force was restored but less than in Group 1 because of the involvement of almost all the contents of the chewing system (Table 4 and Table 5). All patients (Table 5) on chew force (p = 0.001) showed a significant increase in values from postsurgical week one to four. The chew load values also increased significantly from the fourth to sixth postoperative weeks (p = 0.001). From the sixth to the ninth week, the chewing force was also significantly increased.

**Table 4:** Comparison of unilateral mandibular fractures on various postoperative weeks by student's paired t-test POW: postoperative week

POW	Mean	Standard deviation	Mean Difference	Standard Difference	percentage of change	Paired t	P-value
First POW	120.73	4.28					
Fourth POW	151.33	12.79	-30.60	13.22	-25.35	-8.9668	<0.001
First POW	120.73	4.28					
Sixth POW	172.87	16.08	-52.13	14.68	-43.18	-13.7526	<0.001
First POW	120.73	4.28					
Ninth POW	230.00	18.78	-109.27	17.30	-90.50	-24.4592	<0.001
Fourth POW	151.33	12.79					
Sixth POW	172.87	16.08	-21.53	9.91	-14.23	-8.4131	<0.001
Fourth POW	151.33	12.79					
Ninth POW	230.00	18.78	-78.67	20.19	-51.98	-15.0925	<0.001
Sixth POW	172.87	16.08					
Ninth POW	230.00	18.78	-57.13	16.17	-33.05	-13.6859	<0.001

**Table 5:** Comparison of bilateral and unilateral mandibular fractures on various postoperative weeks by student's unpaired t-test POW: Postoperative week, SD: Standard deviation, SE: Standard error

Variable	Fractures	Mean	SD	SE	t-value	P-value
First POW	Bilateral Mandibular	132.73	16.58	4.28	2.7139	0.0113*
	Unilateral Mandibular	120.73	4.28	1.11		
Fourth POW	Bilateral Mandibular	164.33	16.05	4.14	2.4533	0.0206*
	Unilateral Mandibular	151.33	12.79	3.30		
Sixth POW	Bilateral Mandibular	187.60	20.98	5.42	2.1588	0.0396*
	Unilateral Mandibular	172.87	16.08	4.15		
Ninth POW	Bilateral Mandibular	251.27	22.96	5.93	2.7764	0.0097*
	Unilateral Mandibular	230.00	18.78	4.85		
Control	Bilateral Mandibular	262.40	25.24	6.52	2.5413	0.0169*
	Unilateral Mandibular	241.07	20.49	5.29		

## Discussion

A significant relationship between occlusal force and the masticatory system has been explained in many studies. Increased chewing force is a game changer for the action mode on chewing areas [11]. Since increased chew-force levels vary with gender and age, calculations must be standardized along the prefixed values [12-15]. Understanding muscle attachments and the forces imposed upon the mandible will aid the surgeon in the management decisions. It is probably more important to denote simply whether or not a fracture is favorable or unfavorable [16,17]. Mandible fracture and impact may hinder daily activities. Such fractures have a significant impact on chewing and require separate attention from the maxillofacial system [18,19].

This study examined the amount of repair over the chewing system that resulted in a fractured mandible, as well as the risk of mandibular fractures on maximum chewing forces [20-26]. Injury to the region of the face is frequently related to the repair of soft components and maxillofacial structures [27-28]. Inclusion in the treatment of patients with facial trauma involves varieties evaluated for the treatment of facial injuries [29-32]. This feature of fractures significantly impacts the treatment plan. Reduced occlusal forces after surgery may indicate insufficient muscle function; however, maximum bite force in patients can be affected by a variety of other factors, including pain and occlusal derangement following trauma. The molar region has the greatest bite force [33-36].

According to the findings of research that was carried out by Gerlach on patients treated with miniplate osteosynthesis for isolated mandibular angle fractures, the highest vertical loading that was detected in the controls was only 34%. Following the surgical treatment for a fracture, values improved by 59% seven weeks after surgery [37]. Also, according to Throckmorton, significant recovery of increased chew force was noticed in the week of August 6^th^ in cases of both open and closed reduction of mandibular condyle fracture [38]. Any patient's greatest concern is a mandibular fracture and its significant effect on mastication. So, we carried out this comparative study to find out the masticatory forces for traumatic fracture of the mandible patients treated. We can figure out what's going on by looking at the data for the control group, which showed a wide range of bite forces. Interpretation is drawn from data collected for unilateral mandible fracture cases- bite force in the molar region was significantly reduced, albeit temporarily, in the first postoperative week. Restoration of normal architecture is the result of an increase in bite forces, which is gradual and of a small magnitude. A significant difference was found between unilateral mandible fracture cases at various postoperative weeks (Table 4). No significant difference was found between unilateral mandible fracture cases P (0.1343) between the Ninth POW (230.00) and the control group (241.07) (Table 3).

The interpretation that was drawn from the data collected for bilateral mandible fracture cases- normal values approximately equal to those in the control group did not return until the postoperative ninth week. A significant difference is found between bilateral mandibular fracture cases at various postoperative weeks P (0.001) (Table 2). No significant difference is found between bilateral mandibular fracture cases P (0.2168), between the ninth POW mean (251.27), and the control group means (262.40) (Table 1). The mean adult bite force in a unilateral mandible fracture case is 230N. The mean adult bite force in bilateral mandible fracture cases is 251N. Based on the results obtained from our study, we conclude that the mandible fracture affects bite force, although temporarily. Unilateral mandibular fracture cases have a stronger influence on bite force than bilateral mandibular fracture cases. These fractures might prolong stabilization.

## Conclusions

A wide range of bite forces was observed in the control group, and restoration of bite forces in the case of unilateral mandible fracture cases. Bite force in the molar region was significantly reduced in the postoperative first week temporarily, and bite forces in the molar region were close to those of the control group in the post-operative ninth week. Restoration of normal architecture is the result of an increase in bite forces, which is gradual and of a small magnitude. While in bilateral case restoration the normal values were approximately equal to those in the control group, which did not return until the postoperative ninth week. Injury over the face relatively results in trauma to soft tissues, teeth, and major skeletal components of the face, including the mandible, maxilla, zygoma, naso-orbital-ethmoid complex, or supraorbital structures. Participation in the management and rehabilitation of patients with facial trauma involves a thorough understanding of the types of principles of evaluation for and surgical treatment of facial injuries. Whenever facial structures are injured, the goal of the treatment must be maximal rehabilitation of the patient. For facial fractures, goals of treatment include rapid bone healing, a return of normal ocular, masticatory, and nasal function, reconstruction of speech, and an acceptable facial and dental esthetic result. Because of the importance of the mandible as a vital component of the masticatory apparatus, such injuries can be expected to significantly alter occlusion, mandibular range of motion, muscle activity levels, and occlusal forces. Maximum occlusal forces were excellent assessment criteria for restoration of the skeletal architecture and the repair and healing of masticatory soft tissues.
